# Differential modulation of Bax/Bcl-2 ratio and onset of caspase-3/7 activation induced by derivatives of Justicidin B in human melanoma cells A375

**DOI:** 10.18632/oncotarget.21625

**Published:** 2017-10-06

**Authors:** Aljawharah Al-Qathama, Simon Gibbons, Jose M. Prieto

**Affiliations:** ^1^ Centre for Pharmacognosy and Phytotherapy, University College London School of Pharmacy, London WC1N 1AX, United Kingdom; ^2^ Department of Pharmacognosy, Faculty of Pharmacy, Umm Al-Qura University, Makkah 21955, Saudi Arabia

**Keywords:** apoptosis, caspases, Diphyllin, melanoma, cell cycle

## Abstract

Diphyllin and its derivatives are well known cytotoxic natural products structurally related to the anti-cancer drug podophyllotoxin. We here study their structure-activity relationship upon human melanoma cells for first time. To this end, human melanoma A375 cells were incubated with Justicidin B and its 4-methoxylated or 4-glycosylated derivatives to evaluate their selective cytotoxicity and study their effects on cell cycle distribution, caspase activation, apoptosis induction using Annexin V-FITC/PI staining, cell morphology and western blot analysis. Diphyllin methyl ether (GI_50_ = 3.66 μM) and Justicidin B (GI_50_ = 1.70 μM) caused an elevation of both early and late apoptosis populations whereas Diphyllin apioside (GI_50_ = 0.84 μM) and its acetate (GI_50_= 0.39 μM) enhanced late apoptosis population only (Annexin V-positive/PI-positive). All induced cell cycle arrest at S phase and classic morphological indicators of apoptosis (blebbing, apoptotic bodies, and nuclear fragmentation) accompanied with an elevation of cells with low DNA content (sub-G1). All compounds increased the Bax/Bcl-2 ratio by enhancing Bax expression which evidences the involvement of the mitochondria (intrinsic pathway) in the apoptotic process. These caspase-3/7 results evidence that 4-methoxylation or 4-O-glycosylation of Justicidin B -a caspase independent mitochondrial apoptosis-inducer- triggers caspase-3/7 activation at different times (24h vs. 48h, respectively). Interestingly, the methoxylation causes attenuation of Bcl-2 protein expression contrarily to Diphyllin methyl ether or the O-glycosylated derivatives. Finally, the compounds exhibited significantly less toxicity when tested in adult human dermal fibroblasts and their GI_50_ in melanoma Sk-Mel-5 cells was not influenced by MDR1/Pgp inhibitors. This study may inform the synthesis of future Diphyllin derivatives with different apoptosis mechanism of action towards human melanoma cells.

## INTRODUCTION

**Figure 1:**
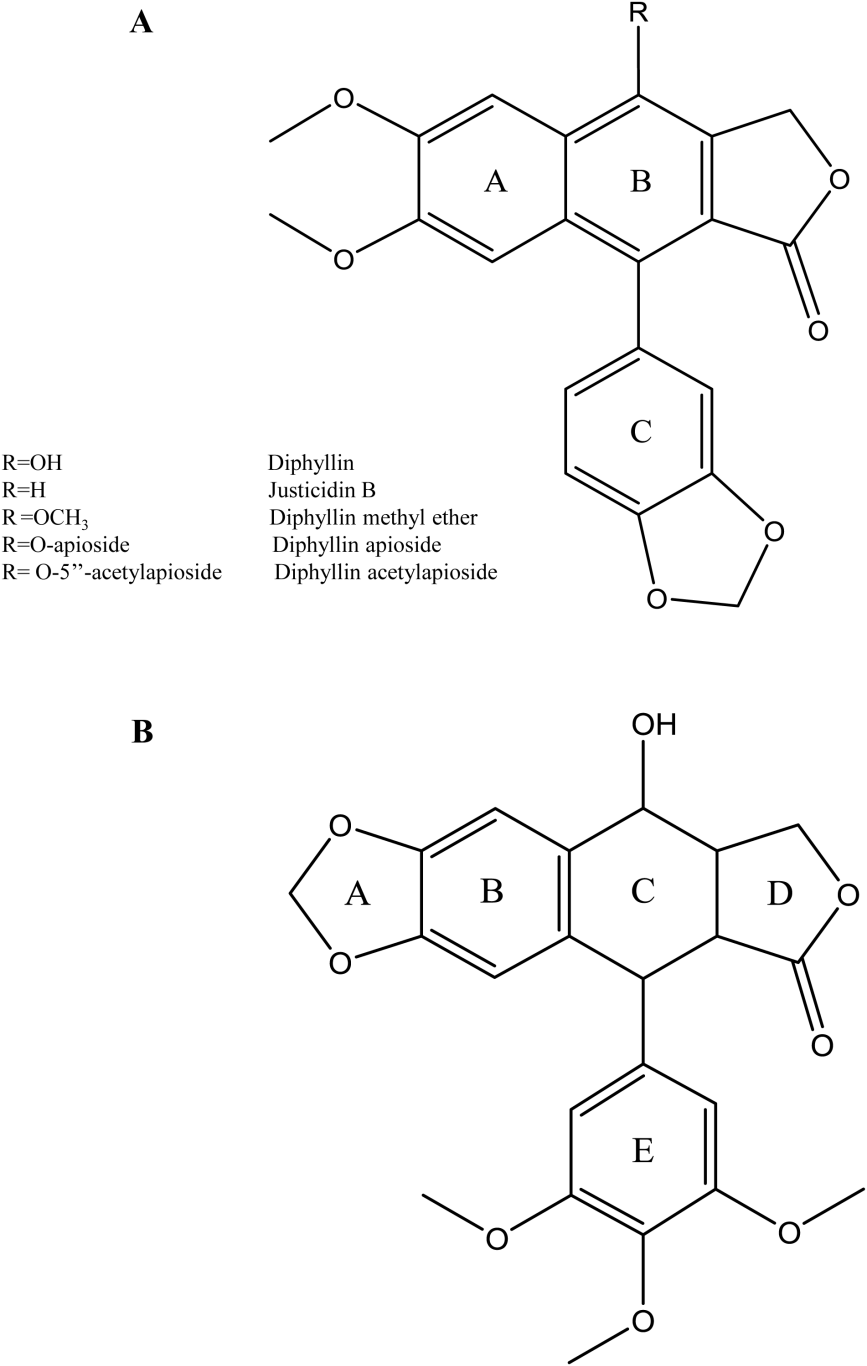
Chemical structures of **(A)** Diphyllin, R= OH; Justicidin B, R=H; Diphyllin methyl ether, R= OCH_3_; Diphyllin apioside, R= O-apioside; Diphyllin acetylapioside, R= O-5’’-acetylapioside, **(B)** Podophyllotoxin.

Justicidin B (Figure 1) is a naturally occurring cytotoxic arylnaphtalene lignan endowed with powerful bioactivities [1]. Its structure is closely related to the well known anti-cancer drug podophyllotoxin and its derivatisation in position 4 gives Diphyllin and other derivatives, which retain the marked cytotoxic activity of the parent molecule. Justicidin B and its derivatives have been isolated from *Haplophyllum tuberculatum* and other species such as *Justicia procumbens* which both have been used traditionally in the treatment of cancer [2, 3].

The cytostatic activities of Diphyllin and some of its derivatives were described in 1979 by González *et al.* [4] who adscribed them to their ability to block the DNA synthesis in both normal and malignant cells pointing to an intercalating action in the minor groove. Later on, the authors claimed that Diphyllin itself have no value as anti-cancer drug, first because its negative cytotoxic index -high tocixicity on both cancer and human primary cells. Modern studies pointed that its anti-proliferative action on cancer cells may involve the cell cycle arrest in the S-phase and inhibition of protein synthesis [5] but also cytotoxic activity towards human monocytes and skin tissues [6] and that it is effluxed by P-glycoprotein (P-gp) [7], thus limiting its therapeutic potential. However, glycosilation may revert the negative cytotoxic index as in the case podophyllotoxin/etoposide. In fact, Cleistanthin A (diphyllin O-(3,4-Di-O-methyl-D-xylopyranoside) is reported to be more toxic to cancer cells than to normal ones [8, 9].

Later work on these class of compounds have reported cytotoxicity mostly at low micromolar range in other cell lines such as human cervical cancer (HeLa 229) [10], human hepatoma (Hep 3B and Hep G2) [11], human colon cancer (HT-29, HCT 116;) and breast cancer (MCF-7) [12] cell lines. Justicidin B was cytotoxic to acute myeloid leukemia (HL-60) [13], breast cancer cell line (MCF-7) [14], human cervical cancer cells (HeLa 229) [10], chronic myeloid leukemia (LAMA-8 and K-562) and chronic lymphoid leukemia (SKW-3) [15] cell lines. Diphyllin apioside, has been reported to have cytotoxic activities against the hepatoma cells (Hep3B), breast cancer cells (MCF-7, MCF-7-ras), human cervical cancer cells (SiHa), human colon cancer cells (HT-29, HCT 116) [16].

Despite all these studies always conclude that Justicidin B is a good lead compound for anti-cancer activity only one attempt to systematically evaluate the structure-activity relationship of its derivatives has been published [17]. The authors conclude that hydroxylation in position 6 of the D-ring enhances cytotoxicity. However, their work analyses the involvement of caspase 3 and the cell cyle distribution at 48h only. Importantly, it does not evaluate their selectivity –i.e cytotoxicity in normal cells- and does not include glycosylated derivatives, which potentially may increase both selectivity and cytotoxicity as already discussed [8, 9]. Despite a number of studies on their *in vitro* and *in vivo* cytotoxic activities on several cancer cell lines, a systematic comparison of the effect of different substitutions upon the mechanism of their apopototic effect remains to be done. Moreover, crude herbal drugs rich in diphyllin derivatives were used since ancient times as topical treatment of warts and pigmentation disorders [18] but to this day –and to the best of our knowledge- there is not any comparative study of their effects upon human melanoma cells or human normal skin cells.

We therefore decided to contribute to a better knowledge of their structure-activity relationship by focusing on the derivatization in position 4 of the B-ring by testing Justicidin B, Diphyllin methyl ether and two naturally occurring glycosylated derivatives (Diphyllin apioside and Diphyllin acetylapioside). Of note, the anti-proliferative activity of the latter has not been previously reported in literature. We used human melanoma cells for first time over an extended period of time (24, 48, and 72h endpoints), compare their effects to those on adult human fibroblasts (48h endpoint) and in addition of caspase-3/7 we investigate their modulation of Bax/Bcl-2 expressions in order to gain further insights into their mechanisms of action.

## RESULTS

### Anti-proliferative activity on human melanoma A375 cells and selectivity towards adult human dermal fibroblasts (HDF-a)

We present in this paper all results in the form of Images and Graphs for convenience. Please download our Supplementary Materials for Tables 1-5 containing the exact numerical results of each experiment.

**Figure 2:**
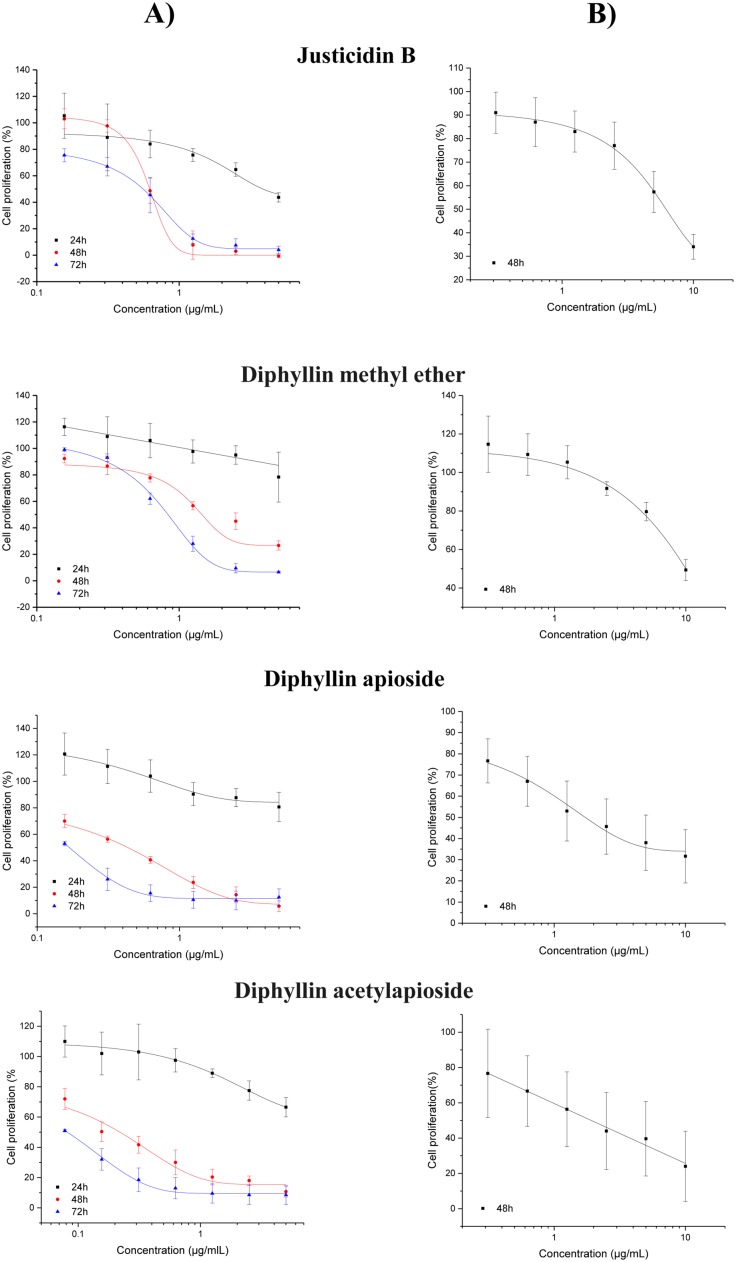
Anti-proliferative effect of Diphyllin and derivatives on **(A)** human melanoma A375 cells and **(B)** adult Human Dermal Fibroblasts (HDF-a) as assessed by the SRB assay (24, 48 and 72h). Results are shown as mean ± SD (n=3).

The inhibitory effects on A375 cell proliferation was assessed by the Sulforhodamine B (SRB) method following cell exposure to several concentrations of Justicidin B and its derivatives at different time points 24, 48 and 72h. Figure 2 shows that all compounds inhibited the proliferation of A375 cells at time and dose-dependent manners as compared to control. The GI_50_ of Justicidin B and Diphyllin methyl ether were found at the low μM range, whilst their glycosylated derivatives at the nM range.

Human dermal fibroblasts have been consistently used as a ‘normal’ cell line closely related to melanoma due to their anatomical proximity and functional relationship, specially the differential ability of fibroblasts to enhance or inhibit the growth of melanoma cells depending on their tumor stage [19, 20]. Figure 2 shows the inhibitory effects on adult human dermal fibroblasts (HDF-a) as assessed by the same method following 48h exposure to the compounds. The relative selectivity of their anti-proliferative effects were 10 fold for Justicidin B (GI_50_ = 17 μM), 6.8 fold for Diphyllin methyl ether (GI_50_ = 25 μM), 3.5 fold for Diphyllin apioside (GI_50_ = 3 μM) and 7.7 fold for Diphyllin acetylapioside (GI_50_ = 3 μM).

### Effects on the human melanoma on A375 cell cycle distribution

**Figure 3:**
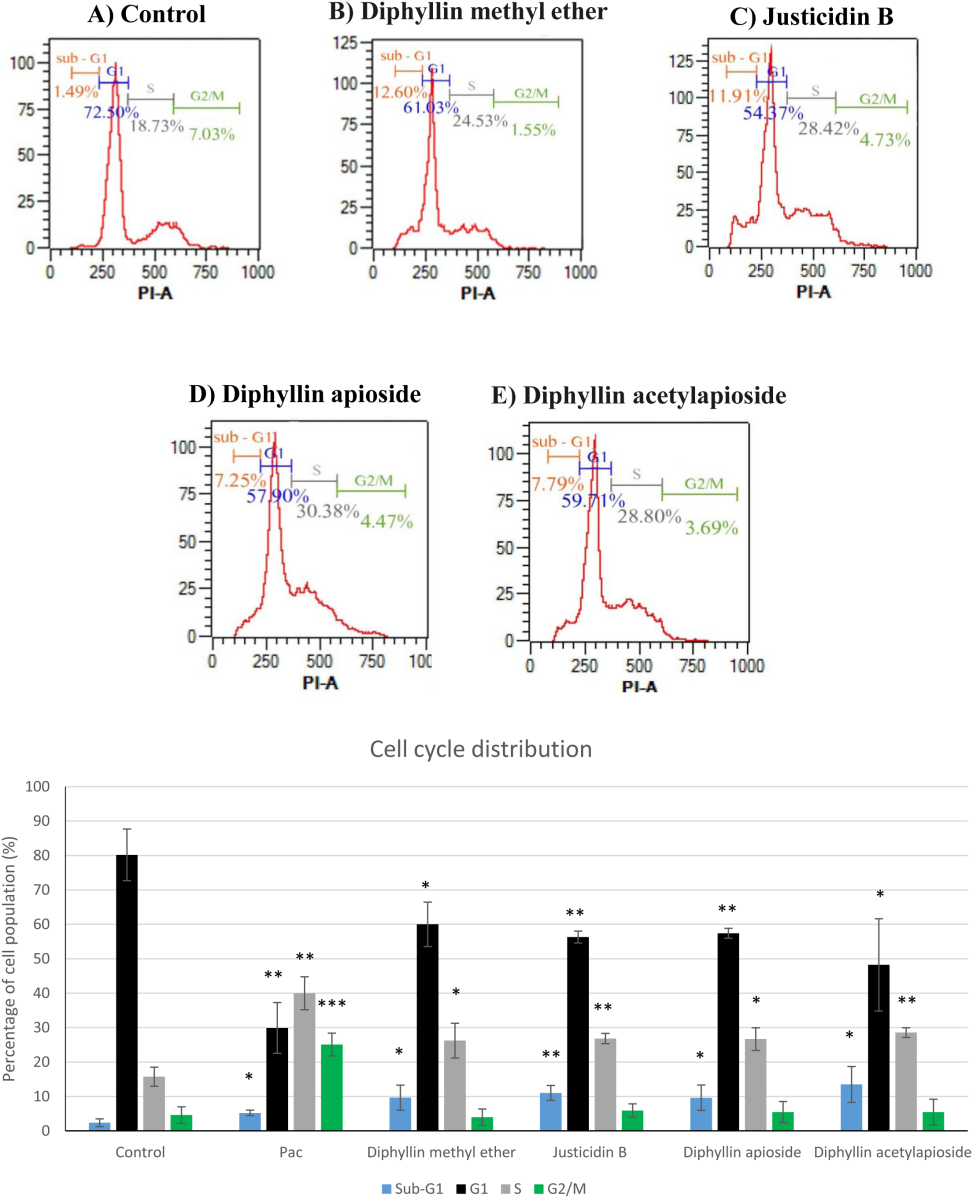
Cell-cycle analysis of human melanoma A375 cells exposed to GI_50_ concentrations of of Diphyllin derivatives at 48h Cell distribution at different stages was analysed by measuring the DNA content using flow cytometry. Data are shown as mean ±SD (n=3). Pac= paclitaxel (positive control).

Quantification of synchronised cells in different phases of the cell cycle at 48h was carried out in A375 cells exposed GI_50_ concentrations of the compounds using flow cytometry to assess whether anti-proliferative activity of Diphyllin derivatives results in a change in cell cycle profile. As shown in Figure 3, the exposure of A375 cells to the compounds resulted in a significant increase of cell population at S phase from 16% in untreated cells to 26-28%. This elevation in S population was associated with a concomitant decrease in G1 population up to about 60%. Moreover, a significant accumulation of cells at sub-G1 peak up to 9-13% was detected in all cases.

### Detection of apoptosis induction by Annexin V- FITC/ PI staining

**Figure 4:**
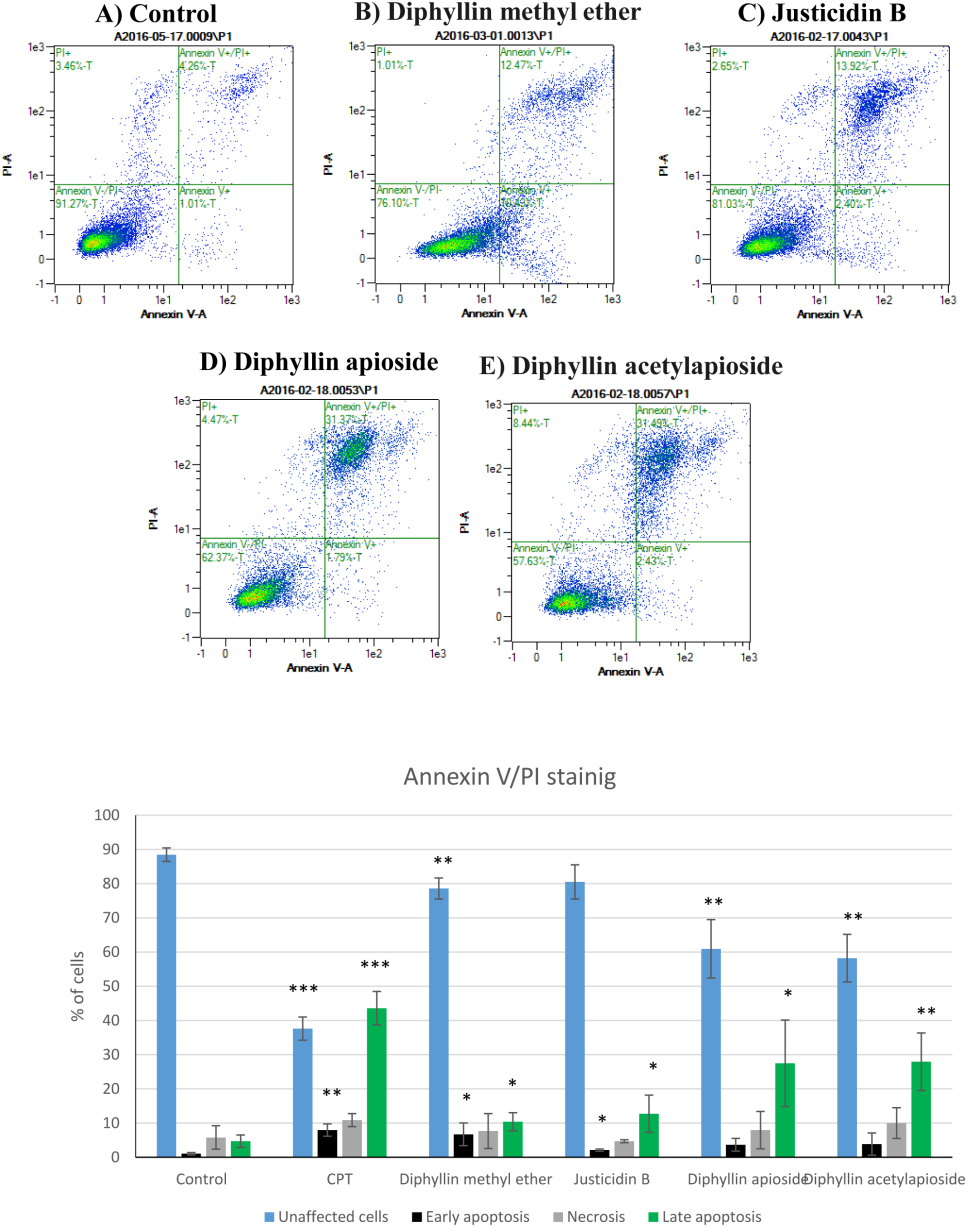
Flow cytometry analysis of human melanoma A375 cells stained with Annexin V-FITC/PI after exposure to GI_50_ concentrations of Diphyllin derivatives during 24h Data are shown as mean ±SD (n=3) of the percentage of unstained, single stained (Annexin-V-FITC or PI) and double stained cell population. CPT= camptothecin (positive control).

Flow cytometry analysis using Annexin V-FITC/PI double staining was performed to assess the translocation of phosphatidylserine to the outer leaflet of cell membrane -a marker of early apoptotic cells [21]- following cell exposure to Diphyllin derivatives. Figure 4 shows that 24h treatment with Justicidin B treatment of A375 cells at resulted in a significant enhancement in both early (Annexin V-positive/ PI-negative, 2.10%, *p* = 0.0178) and late apoptotic populations (Annexin V-positive/ PI-positive, 12.71%, *p* = 0.0435). Diphyllin methyl ether markedly increased early apoptotic cells 6.70% compared to control 1.03% (*p* = 0.0420). The total percent of late apoptotic cells was also elevated from 4.22% up to 10.38% (*p* = 0.0373) and the number of viable cells was significantly reduced (78.56% *p* = 0.0096) in comparison to untreated cells (88.46%) without a significant change in the number of PI-positive population.

The percentage of viable cells was significantly reduced to 60.93% (*p* = 0.0055) in Diphyllin apioside-treated cells and up to 58.22% (*p* = 0.0019) following cell exposure to Diphyllin acetylapioside. The effects of Diphyllin apioside and Diphyllin acetylapioside were accompanied with a significant elevation in the percentage of late apoptotic population (Annexin V-positive/ PI-positive cells) reaching 27.46% (*p* = 0.0366) and 27.92% (*p* = 0.0095). Less than 10% of population showed necrotic signs (Annexin V-negative/ PI-positive) after exposure to all compounds. Shorter incubations (12h) did not show any sign of early apoptosis (See Supplementary Figure 1 in Supplementary Materials).

### Morphological changes induced by Diphyllin derivatives on A375 cells

**Figure 5:**
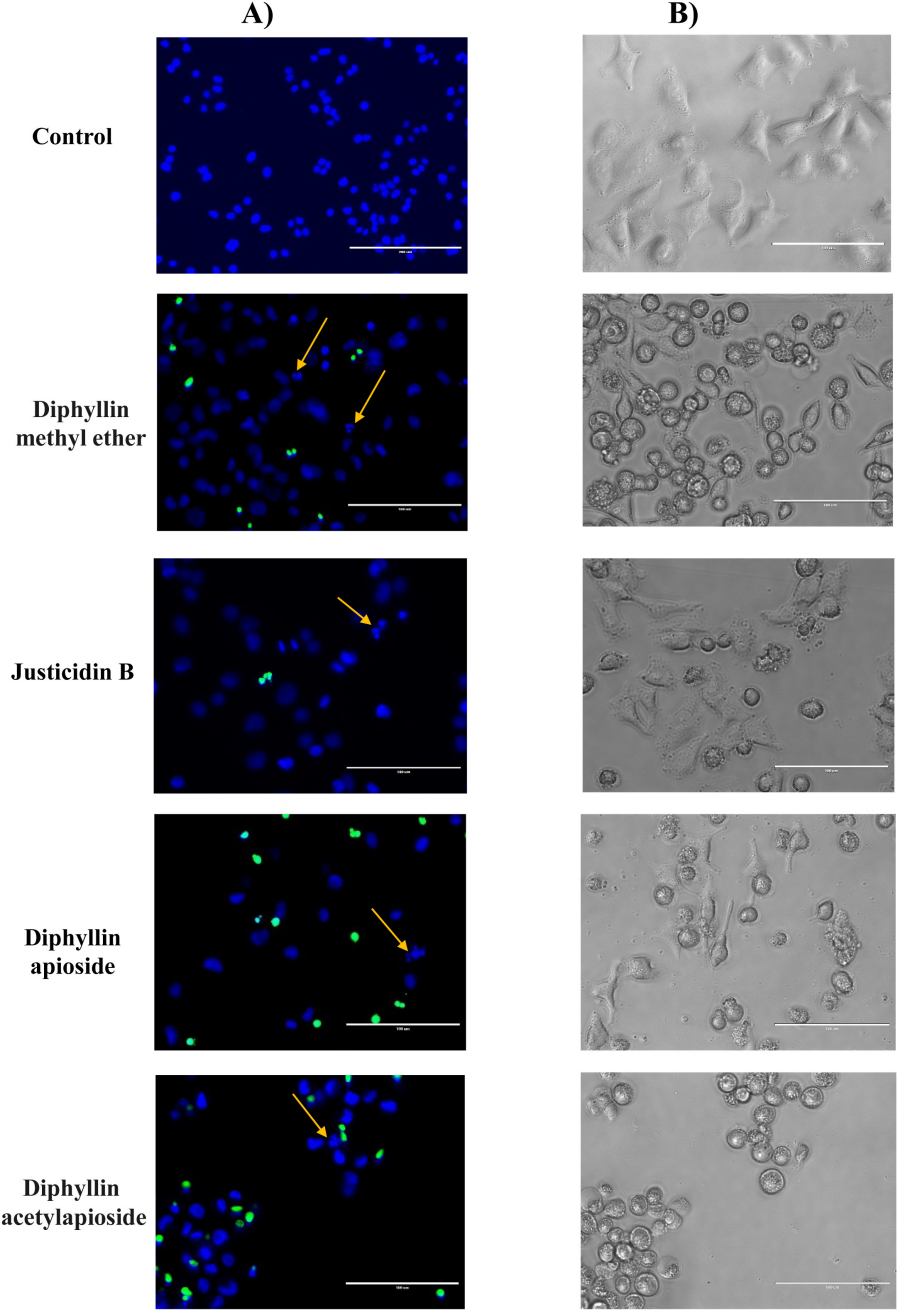
Microscopy analyses of human melanoma A375 cells after exposure to GI_50_ concentrations of Diphyllin derivatives during 48h **(A)** Live cell micrographs (fluorescence, 40x) of cells stained with Hoechst 33342 and NucGreen^®^ Dead reagent (arrow indicates nuclear fragmentation). **(B)** Morphological changes observed (phase contrast microscope, 40x). Bar = 100 μm.

The viability of A375 cells treated with GI_50_ concentrations of Diphyllin derivatives were qualitatively evaluated using phase contrast microscope and double staining with Hoechst 33342 and NucGreen^®^ -that stains cells with compromized membranes. The results shown in Figure 5 confirmed those obtained from SRB assay: in contrast to control cells, cells treated with any of the Diphyllin derivatives showed a shrinkage in size, round in shape and disruption of cells attachment accompanied with characteristic features of apoptotic death such as formation of apoptotic bodies and membrane blebbing and nuclear fragmentation as revealed by a strong blue fluorescence.

### Differential onset of caspase-3/7 activity after treatment with Diphyllin derivatives

**Figure 6:**
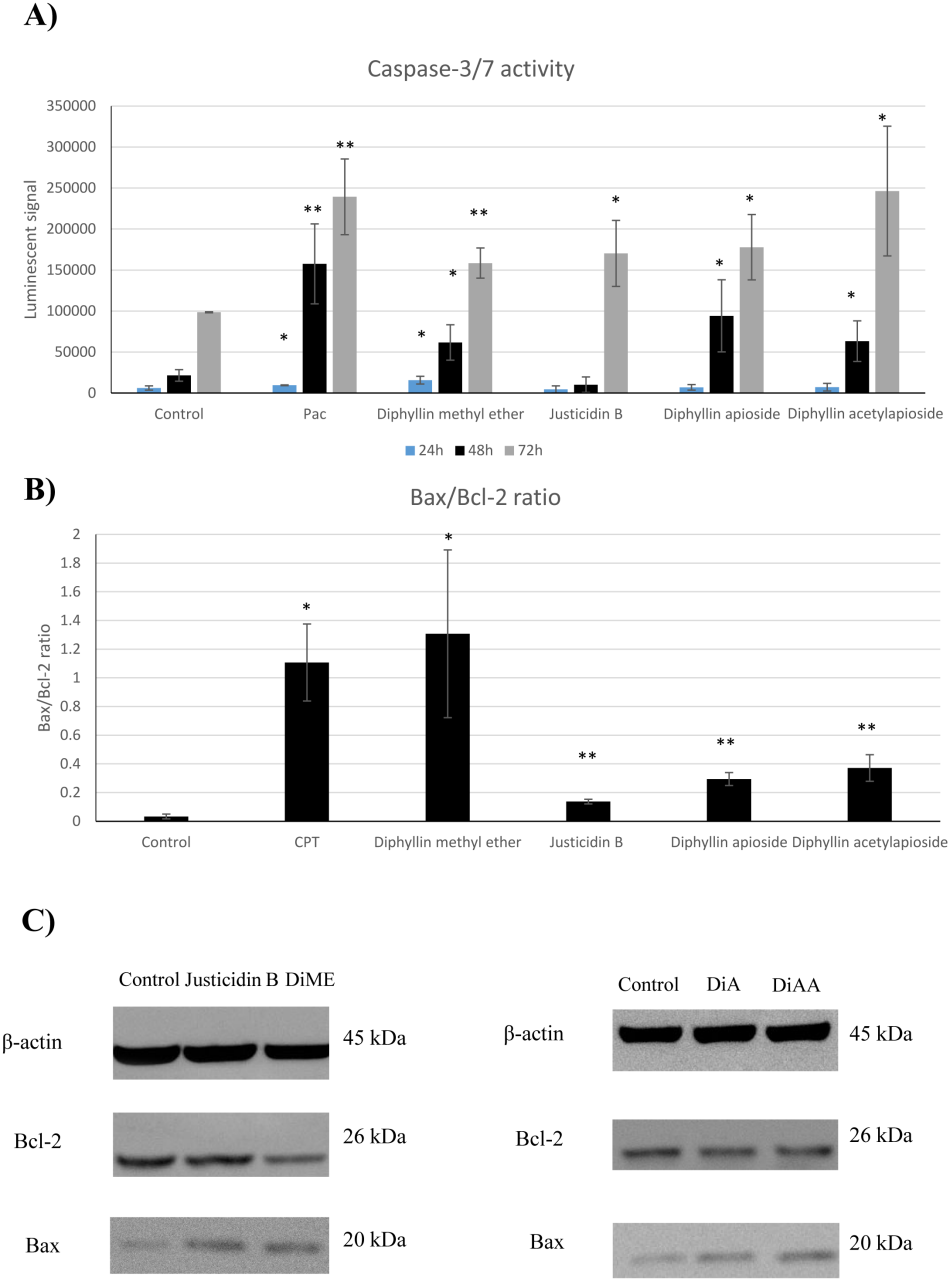
**(A)** Caspase-3/7 activity in human melanoma A375 cells treated with GI_50_ of Diphyllin and derivatives at different incubation periods (24, 48 and 72h). Pac= paclitaxel (positive control). **(B)** Densitometric evaluation of the expression levels of Bax/Bcl-2 proteins normalised to β-actin and changes in Bax/Bcl-2 ratio, CPT= camptothecin (positive control). **(C)** Immunoblot images of their expression in human melanoma A375 cells after 48h treatment with GI_50_ of Justicidin B, Diphyllin methyl ether (DiME), Diphyllin apioside (DiA), and Diphyllin acetylapioside (DiAA). Data are presented as the mean ±SD (n=3).

In an attempt to assess whether the inhibitory effects on cell proliferation associated with effects on caspase-mediated apoptosis pathway, caspase-3/7 activity was measured. As shown in Figure 6A, Diphyllin methyl ether induced significantly the activity of caspase-3/7 in a time-dependent manner following 24, 48 and 72h exposure by 2.67 fold (*p* = 0.0297), 2.85 fold (*p* = 0.0375) and 1.60 fold (*p* = 0.0048), respectively. However, in case of Justicidin B treatment there was no significant change detected at 24, 48h periods whereas the activity of caspase-3/7 was significantly induced after 72h treatment by 1.72 fold (*p* = 0.0366). The results indicated that there was no difference between untreated and treated cells with glycosylated derivatives after 24 treatment but it was significantly induced within 48h exposure to Diphyllin apioside and Diphyllin acetylapioside by 4.36 fold (*p* = 0.0475) and 2.93 fold (*p* = 0.0351), respectively. Similar effect was detected at 72h incubation period reaching 1.8 fold (*p* = 0.0260) for Diphyllin apioside and 2.49 fold (*p* = 0.0318) for Diphyllin acetylapioside treatments.

### Bax and Bcl-2 expression in the presence of different Diphyllin derivatives

In this work, we aim to further gain insight into the expression of Bcl-2 family core members, Bax and Bcl-2, and assess the mitochondrial activation caused by these compounds after A375 cells were exposed for 48h to GI_50_ concentrations of Diphyllin derivatives. As shown in Figure 6C, densitometry of the distinctive bands corresponding to Bax (20 kDa) and Bcl-2 (26 kDa) demonstrated a significant increase in the protein expression of Bax after treatment with Diphyllin methyl ether (*p* = 0.0001) normalised to β-actin (45 kDa). Contrary to the increase of the Bax expression, the level of the anti-apoptotic factor Bcl-2 was markedly reduced (*p* = 0.0470). This resulted in a significant elevation of the ratio of Bax/Bcl-2 (*p* = 0. 0196) compared to that of the control in favour of apoptosis in A375 cells. However, the examination of those exposed to justicidin B showed no significant changes in expression of Bcl-2 whereas the level of Bax was markedly elevated (*p* = 0.0065) in comparison to their controls, therefore the ratio of Bax/Bcl-2 was significantly increased (*p* = 0.0016).

In cells treated with Diphyllin apioside and Diphyllin acetylapioside, the expression of Bax protein was significantly upregulated (*p* = 0.0090) and (*p* = 0.0378), respectively. Interestingly, the level of Bcl-2 remained the same as untreated cells, however Bax/Bcl-2 ratio was markedly changed (*p* = 0.0079, Diphyllin apioside) and (*p* = 0.0037, Diphyllin acetylapioside) demonstrating the elevation of Bax/Bcl-2 ratio.

### Cytotoxicity of the studied Diphyllin derivatives is not affected by inhibitors of the MDR1/Pgp

Previous reports pointed towards Diphillyn as a substrate of P-glycoprotein (MDR1/P-gp) [7], thus potentially limiting its therapeutic potential. This prompted us to test their *in vitro* cytotoxicity on the melanoma cell line SK-Mel-5, reported to be one of the melanoma lineages expressing higher levels of the ABCB1 transcript [22], both alone and in the presence of non toxic concentrations of verapamil, a known Pgp inhibitor widely used in cancer research [23]. The results are presented in Table 1. In line with the data from Szakács *et al.* [22]: treatment of Sk-Mel-5 cells with Vincristine, a cytotoxic drug known to be effluxed by MDR1/Pgp is predictably affected by co-treatment with 2.3 μM Verapamil, whilst Camptothecin did not. The diphyllin derivatives did not have their GI_50_ values significantly affected by the co-treatment.

**Table 1:** Values of growth inhibition 50% of reference cytotoxic drugs and diphyllin derivatives in Sk-mel-5 cells co-incubated with or without the MDR1/Pgp inhibitor verapamil (*n* = 3)

	+ vehicle	+ verapamil (2.3μM)
Vincristine	1.04±0.90 nM	0.15±0.02 nM (p <0.01)
Camptothecin	6.93±1.51 nM	7.22±2.15 nM
Diphyllin methyl ether	2.33±0.24 μM	2.49±0.51 μM
Justicidin B	1.15±0.26 μM	1.22±0.23 μM
Diphyllin apioside	0.050±0.016 μM	0.046±0.045 μM
Diphyllin acetylapioside	0.045±0.008 μM	0.040±0.015 μM

## DISCUSSION

Justicidin B and the three tested Diphyllin 4-derivatives significantly suppress the proliferation of human melanoma A375 cells in a time and concentration dependent manner. The antiproliferative activity of these compounds seems not to be influenced by MDR1/Pgp efflux as per our results in a functional assay (Table 1). Justicidin B induces an elevation in sub-G1 population within 48h with all morphological changes associated with apoptosis (blebbing, apoptotic bodies, nuclear fragmentation and externalization of phosphatidylserine). Activation of caspase-3/7 is evident only after 72h, thus pointing towards an initial caspase-independent mechanism of action driven by the elevated Bax levels after 48h that tilt the Bax/Bcl-2 ratio towards the release of pro-apoptotic molecules from mitochondria. Methoxylation in position 4 changes the dynamics by which Justicin B triggers apoptosis in A375 melanoma cells. The methyl ether derivative exerts the same effects of the parent molecule –albeit at a marginally higher GI_50_ (3.66 μM vs. 1.70 μM)- with a significant activation of caspase-3/7 within 24h, and a reduction of Bcl-2 expression which further increases the Bax/Bcl-2 ratio.

Phosphatidylserine externalization and DNA fragmentation may occur independently of caspase action [24]. Luo's group has reported that justicidin B translocated phosphatidylserine to the outer membrane of the human leukemia cell line (K562) indicating early apoptosis similar to A375 cells in this study [17]. Similar effect for Diphyllin methyl ether was demonstrated in other reports in which the proportion of apoptotic cells with externalized phosphatidylserine was elevated in human colorectal cancer cells (HT-29 and HCT 116) [12] and human hepatoma cells [11]. Whether caspase-independent death effectors such apoptosis-inducing factor (AIF) [25] and endonuclease G [26] may or not be activated by this compound warrant further attention. But caspase dependent mechanism was clearly implicated in the DNA fragmentation in MCF-7 and MDA-MB-231 breast cancer after 24h as this proapoptotic activity was completely inhibited by the non-selective pan-caspase inhibitor Boc-Asp(OMe)-fluoromethyl ketone. However, Justicidin B exerted different effects in the two cell lines increasing and decreasing the expression of NF-κB, respectively. This highlighting that Justicidin B may act by different mechanisms depending on the cancer cell line no matter how similar they are. A robust activation of caspase-3/7 by Diphyllin methyl ether in other cell lines was similarly reported by Won *et al*, in human hepatoma cells [27] but other caspases have been reported to be activated in response to Diphyllin methyl ether treatment such as caspase-8 in human hepatoma Hep 3B and Hep G2 cells [11]. In addition, caspase-9 was activated in HT-29 and HCT 116 colorectal cancer cells, eventually leading to translocation of Bax from the cytosol to mitochondria to induce apoptosis [12].

The glycosilation of the hydroxyl in position 4 with apioside, a bulkier, more polar group, brings about a sizeable reduction of the GI_50_ down to nM ranges but delays activation of caspase-3/7 with respect to Diphyllin methyl ether. Further acetylation of the apioside halves the GI_50_ of the glycosylated Diphyllin and improves its selectivity against normal cells by 8 fold.

This study reiterates that the anti-proliferative activity of Diphyllin derivatives linked to a pronounced S phase arrest. As DNA is replicated once during the S-phase, the arrest of cell distribution at this phase results in the status where cells neither go forward nor retreat to G1 stage [28]. Moreover, the first symptom of apoptosis induction by these compounds is the accumulation of cells at sub-G1 peak which is considered as a biomarker for DNA fragmentation to low molecular weight in which cells manifest with a reduced DNA content. Diphyllin methyl ether, also caused elevations in the sub-G1 fraction in human hepatoma cells [11], colon colorectal cells [12]. In addition, Justicidin B has shown in literature an increase in the level of the apoptotic DNA fragmentation in human leukemia cell lines (HL-60) [13] and K562 [17].

As previously mentioned in the introduction, Diphyllin has been traditionally regarded as having a negative selectivity. However, in the case of Justicidin B there was only one study evaluating the selectivity of this lignan against Jurkat cells and PBMC where a nonspecific cytotoxicity was detected [29]. In our hands, it displayed at least 10 fold higher GI_50_ to HDF-a cells than the GI_50_ to human melanoma A375 cells. The GI_50_ concentration of Diphyllin methyl ether towards HDF-a was 7 fold higher compared to the GI_50_ for cancer cells thus showing similar selectivity to paclitaxel (positive control). Su *et al*., have reported similar effect where higher GI_50_ was about 10 fold towards non-malignant Chang liver cells [11]. Moreover, Diphyllin methyl ether had low toxicity in normal human peripheral blood mononuclear cells (PBMC) and human embryonic kidney cells (HEK293) up to 500, 100 fold, respectively, compared to cancer cells [12, 30]. Diphyllin acetylapioside had two times (8 fold) more selectivity compared to the parent molecule (4 fold).

Bcl-2 family is a crucial mediator of mitochondria-dependent pathway controlling mitochondrial outer membrane permeabilization through the interactions between pro-apoptotic (Bax) and anti-apoptotic (Bcl-2) core members [31, 32]. The higher Bax/Bcl-2 ratio is, the high is the cell susceptibility to apoptosis which is another indicator that Diphyllin methyl ether induced cellular apoptosis by targeting the intrinsic pathway. The explanation of how Diphyllin methyl ether impaired the mitochondria at 48h exposure could be that Bcl-2 protein is down regulated and thus unable to control Bax which was up-regulated. Consequently, transition pores of mitochondrial membrane increase in size and become large enough allowing cytochrome *c* to be released from the mitochondria which is essential for caspase activation [32]. In agreement with these findings, Lee *et al.*, and Su *et al.*, have mentioned the ability of Diphyllin methyl ether to reduce Bcl-xL expression and increase Bax and Bak level in hepatoma and colorectal cancer cells [11, 12]. *In vivo* studies have been conducted showing the oral administration of Diphyllin methyl ether suppressed the growth of hepatoma cells and colorectal cancer cells implanted in NOD-SCID mice [11, 12].

In cells treated with Justicidin B, Diphyllin apioside and Diphyllin acetylapioside, only Bax protein expression was increased after 48h exposure and such an effect on Bcl-2 family has not been reported previously. Involvement of Bax protein in the mechanism of action of these lignans reveals the role of mitochondrial impairment in cell death and that was detected with or without the involvement of caspase, as mentioned above for Diphyllin apioside and Diphyllin acetylapioside as well as justicidin B, respectively. Bax has been reported to have a direct effect on both caspase dependent and independent pathways in which Bax activation and its translocation from cytosol to mitochondria triggers the release of cytochrome *c*, which is essential for caspase activity, AIF and endonuclease G causing DNA fragmentation and chromatin condensation [33] which could explain how these compounds works as pro-apoptotic agents in A375 cells.

In summary, we here evidence that 4-methoxylation or 4-O-glycosylation of Justicidin B -a caspase independent mitochondrial apoptosis-inducer- differently triggers the onset of caspase-3/7 activation (24h vs. 48h, respectively). Interestingly, the methoxylation causes attenuation of Bcl-2 protein expression contrarily to the parent molecule or the O-glycosylated derivatives. If we take together our results and those from Luo's structure-activity relationship study [17], there is a a possibility to dramatically increase the cytotoxicity of Justicidin B when this is glycosylated at position 4 of ring B and hydroxylated at position 6 of ring D. These considerations may inform future synthesis of Diphyllin derivatives with improved cytotoxicity and selectivity towards cancer cells.

## MATERIALS AND METHODS

### Chemicals

Diphyllin methyl ether (Justicidin A), Justicidin B, Diphyllin apioside (tuberculatin) and Diphyllin acetylapioside (tuberculatin acetate) were isolated from *Haplophyllum tuberculatum* as previously described [34]. The following were purchased from Sigma-Aldrich (St. Louis, MO, USA): Sulforhodamine B, trichloroacetic acid, Trizma base, propidium iodide (PI), Ribonuclease A (RNase). Glacial acetic acid, ethanol, methanol and chloroform were obtained from Fisher (Leicestershire, UK). TrypLE Express (1×, trypsin, EDTA, phenol red), phosphate-buffered saline (PBS) and trypan blue were purchased from Thermo Fisher Scientific (MA, USA).

### Cell lines

The cytotoxicity and the selectivity of all compounds were evaluated using A375 and SK-Mel-5 human melanoma cell (ATCC, American Type Culture Collection, Manassas, VA, USA) and human dermal fibroblasts (HDF-a, Thermo Fisher Scientific, MA, USA), respectively. The medium used to maintain A375 cells was Dulbecco's Modified Eagle's Medium (DMEM, Gibco) with 10% heat-inactivated fetal bovine serum (FBS, Gibco) and 1% of antibiotic solution (10,000 U/mL, Penicillin-Streptomycin, Gibco). HDF-a cells were grown in MEM supplemented with 10% FBS, 1% MEM non-essential amino acids solution (NEAA, Gibco) and Gentamicin/Amphotericin Solution (5 mg/mL Gentamicin, 125 μg/mL Amphotericin B, Gibco). SK-Mel-5 cells were maintained in Eagle's Minimum Essential Medium (ATCC, VA, USA) with 10% FBS and 1% of antibiotic solution (10,000 U/mL, Penicillin-Streptomycin). All cell lines were cultured in complete growth medium (10% FBS) and incubated in an incubator with humidified air 5% CO_2_ and atmosphere at 37°C.

### Cell proliferation assay

The anti-proliferative activity of the compounds in tumor and normal cells was assessed by the sulforhodamine B (SRB) assay as previously described [35]. Cells were seeded into 96-well plates in the density of 1×10^4^/well and incubated overnight. After 24h, medium was removed and replaced with complete growth medium supplemented with different concentrations of the compounds for 24, 48 and 72h then assayed for growth inhibition using SRB method. Adherent cells were fixed by the addition of cold 40% (w/v) trichloroacetic acid to achieve a final concentration of 10% and incubated for 1h at 4°C. The plates were washed with deionised water and SRB solution (4 % SRB in 0.1% acetic acid) was added and incubated for 30 mins at room temperature. Unbounded stains were removed by washing plates with 1% acetic acid. The bounded stain was later solubilized with 10 mM Trizma base buffer and absorbance was measured (Infinite^®^ M200, Tecan, Switzerland), data normalized to untreated wells, GI_50_ value was calculated as the concentration that results in 50% cell growth inhibition and graphs were drawn on OriginPro software (version 9.3.1.273).

### Cell cycle analysis

Cell cycle distribution was analysed by measuring the DNA content using flow cytometry as previously mentioned [36]. When A375 cells reach 70% confluence, they were plated at low-serum medium (2%FBS) at a density of 2.5×10^5^ cells/well in 6-well plates. After 24h, they were incubated with the compounds in complete growth medium for 48h. Then, cells were harvested by trypsinization and also the floating cells in the medium was collected. They were pelleted by centrifugation, washed twice with PBS, resuspended in ice-cold 70% ethanol in PBS for fixation and kept at 4°C for 24h. Prior analysis, fixed cells were washed with PBS and incubated with PI staining solution containing 100μg/mL of RNase and 50μg/mL of PI in PBS in the dark for 30 mins. Cellular DNA content was analysed by FACS analysis using The MACSQuant^®^ Analyzer 10 and MACSQuantify software (Miltenyi Biotec, Germany) for cell cycle analyses. The results were expressed as the percentage of cell population in each phase (mean ± SD, n = 3).

### Flow cytometry analyses for apoptosis induction

Analysis of the translocation of phosphatidylserine to the outer leaflet of cell membrane was performed according to the manufacturer's instructions (Annexin V-FITC Kit, Miltenyi Biotec, Germany). Briefly, 1×10^5^ A375 cells were seeded in 12-well plates overnight and treated with the compounds for 24h. Cells were washed with PBS and the binding buffer and then resuspended in the binding buffer containing 10 μL of Annexin V-FITC for 15mins in the dark. After washing the cells with binding buffer again, they were incubated with 1μg/mL of PI in binding buffer prior to analysis by FACS. Data acquisition was performed using The MACSQuant^®^ Analyzer 10 and MACSQuantify software (Miltenyi Biotec, Germany) for data analysis. Data was shown as the percentage of unstained/single or double stain cells in four populations (mean ± SD, n = 3).

### Cellular morphology by phase contrast microscope and cell viability imaging by fluorescence microscope

Morphological assessment of A375 cells was performed to detect the cellular changes induced by the tested compounds according to the method with slight modifications [37]. Briefly, 1×10^4^ cells were treated with or without the compounds in a sterile chamber slide system (Nunc™ Lab-Tek™ II Chamber Slide™ System) for 48h. Prior cellular examination, the media was removed and replaced with PBS. Treated cells with typical morphological changes of apoptosis were imaged using phase contrast inverted microscope (EVOS cell imaging system, Thermo Fisher Scientific, MA, USA).

A375 cells were examined with fluorescence microscope using ReadyProbes^®^ cell viability imaging kit according to manufacturer's recommendations (Thermo Fisher Scientific, MA, USA). A375 cells were treated in the same condition as mentioned in the previous section. Both dyes were added for 15 mins and slides were visualised using EVOS cell imaging system (Thermo Fisher Scientific).

### Caspase-3/7 activity assay

Luminescent caspase-Glo 3/7 assay was carried out on exposed cells to Diphyllin and its derivativesaccording to the manufacturer's instructions (Promega, WI, USA). A375 cells were seeded in white 96-well plates and treated with the cytotoxic compounds for 24, 48 and 72h. The cells were incubated with caspase-Glo 3/7 reagent for 1 h at room temperature which was followed by measuring the enzymatic activity of caspase through luminescent signal using microplate reader (Infinite^®^ M200, Tecan).

### Western blot analysis

Whole-cell extracts were prepared and analysed by Western blot kit according to manufacturer's recommendation (Cell Signaling Technology, MA, USA). After A375 cells were treated with the Diphyllin for 48h, they were lysed with lysis buffer and sonicated and then the cell extract was centrifuged. The protein concentration was quantified using BCA kit (Cell Signaling Technology) as described by the manufacturer. The protein extracts (50μg) were suspended in the loading buffer after heating to 95°C. They were loaded to standard SDS-PAGE (12% Tris-Glycine, BioRad, Herts, UK), along with prestained protein marker (11-190 kDa) and biotinylated protein ladder to detect the molecular weight ladders and electrophoresis was carried out at 110 for 95 mins (PowerPac™ Basic power Bio-Rad, California, USA). The gel was transferred onto nitrocellulose membrane and then it was blocked in blocking buffer, 5% non-fat dry milk in 1X Tris Buffered Saline with Tween 20. This was followed by probing the membrane with Bax, Bcl-2 and β-actin proteins (Cell Signaling Technology, MA, USA), the later was used as a standard for the amount of the loaded protein (50μg) in each lane. The bound primary antibody complex and biotinylated protein marker were then stained using horseradish peroxidase-conjugated anti-mouse, anti-rabbit, and anti-biotin secondary antibodies. Protein bands were detected with enhanced chemiluminescence reagents and visualised by GeneGnome for chemiluminescence imaging (Syngene Bio Imaging, Frederick, MD, USA) using GENESys (version 1.3.3.0). Data was expressed as the ratio of Bax and Bcl-2 that was normalized to β-actin (n = 3). Densitometric evaluation of the percent change in expression levels of Bax/Bcl-2 proteins was carried out using GelQuantNET (version 1.8.2).

### Statistical analysis

Results are expressed as mean ± standard deviation (SD) from at least three independent experiments. All data were analysed using unpaired two-tailed Student's *t*-test with a *p* value of <0.05 considered as significant to find the statistical significance between treated groups and controls.

## SUPPLEMENTARY MATERIALS FIGURE AND TABLES



## References

[1] Hemmati S, Seradj H (2016). Justicidin B: a promising bioactive lignan. Molecules.

[2] Zhou P, Luo Q, Ding L, Fang F, Yuan Y, Chen J, Zhang J, Jin H, He S (2015). Preparative isolation and purification of lignans from Justicia procumbens using high-speed counter-current chromatography in stepwise elution mode. Molecules.

[3] Raissi A, Arbabi M, Roustakhiz J, Hosseini M (2016). Haplophyllum tuberculatum: an overview. J HerbMed Pharmacol.

[4] Gonzalez AG, Darias V, Alonso G (1979). Cytostatic lignans isolated from Haplophyllum hispanicum. Planta Med.

[5] Di Giorgio C, Delmas F, Akhmedjanova V, Ollivier E, Bessonova I, Riad E, Timon-David P (2005). vitro antileishmanial activity of diphyllin isolated from Haplophyllum bucharicum. Planta Med.

[6] Schinella G, Tournier H, Zaidenberg A, Prieto JM (2008). On the preclinical anti-trypanosomal, anti-inflammatory and toxicological activities of H. linifolium (L.) G. Don and its diphyllin derivatives. Bol Latinoam Caribe Plant Med Aromat.

[7] Lim S, Grassi J, Akhmedjanova V, Debiton E, Balansard G, Beliveau R, Barthomeuf C (2007). Reversal of P-glycoprotein-mediated drug efflux by eudesmin from Haplophyllum perforatum and cytotoxicity pattern versus diphyllin, podophyllotoxin and etoposide. Planta Med.

[8] Pradheepkumar CP, Panneerselvam N, Shanmugam G (2000). Cleistanthin A causes DNA strand breaks and induces apoptosis in cultured cells. Mutat Res.

[9] Pradheepkumar CP, Shanmugam G (1999). Anticancer potential of cleistanthin A isolated from the tropical plant Cleistanthus collinus. Oncol Res.

[10] Gonzalez AG, Darias V, Alonso G (1979). Cytostatic lignans isolated from Haplophyllum hispanicum. Planta Medica.

[11] Su CL, Huang LL, Huang LM, Lee JC, Lin CN, Won SJ (2006). Caspase-8 acts as a key upstream executor of mitochondria during justicidin A-induced apoptosis in human hepatoma cells. FEBS Lett.

[12] Lee JC, Lee CH, Su CL, Huang CW, Liu HS, Lin CN, Won SJ (2005). Justicidin A decreases the level of cytosolic Ku70 leading to apoptosis in human colorectal cancer cells. Carcinogenesis.

[13] Momekov G, Yossifov D, Guenova M, Michova A, Stoyanov N, Konstantinov S, Ionkov T, Sacheva P, Ionkova I (2014). Apoptotic mechanisms of the biotechnologically produced arylnaphtalene lignan justicidin B in the acute myeloid leukemia-derived cell line HL-60. Pharmacol Rep.

[14] Momekov G, Konstantinov S, Dineva I, Ionkova I (2011). Effect of justicidin B – a potent cytotoxic and pro-apoptotic arylnaphtalene lignan on human breast cancer-derived cell lines. Neoplasma.

[15] Vasilev N, Yaman E, Bos R, Kayser O, Momekov G, Konstantinov S, Ionkova I (2006). Production of justicidin B, a cytotoxic arylnaphthalene lignan from genetically transformed root cultures of Linum leonii. J Nat Prod.

[16] Day SH, Lin YC, Tsai ML, Tsao LT, Ko HH, Chung MI, Lee JC, Wang JP, Won SJ, Lin CN (2002). Potent cytotoxic lignans from Justicia procumbens and their effects on nitric oxide and tumor necrosis factor-α production in mouse macrophages. J Nat Prod.

[17] Luo J, Hu Y, Kong W, Yang M (2014). Evaluation and structure-activity relationship analysis of a new series of arylnaphthalene lignans as potential anti-tumor agents. PLoS One.

[18] Prieto JM, A Fischl S (2011). Haplophyllum A. Juss, A Rich Source of Bioactive Natural Principles. Bitterlich.

[19] Cornil I, Theodorescu D, Man S, Herlyn M, Jambrosic J, Kerbel RS (1991). Fibroblast cell interactions with human melanoma cells affect tumor cell growth as a function of tumor progression. Proc Natl Acad Sci U S A.

[20] Philips N, Keller T, Hendrix C, Hamilton S, Arena R, Tuason M, Gonzalez S (2007). Regulation of the extracellular matrix remodeling by lutein in dermal fibroblasts, melanoma cells, and ultraviolet radiation exposed fibroblasts. Arch Dermatol Res.

[21] Elmore S (2007). Apoptosis: a review of programmed cell death. Toxicol Pathol.

[22] Szakacs G, Annereau JP, Lababidi S, Shankavaram U, Arciello A, Bussey KJ, Reinhold W, Guo Y, Kruh GD, Reimers M, Weinstein JN, Gottesman MM (2004). Predicting drug sensitivity and resistance: profiling ABC transporter genes in cancer cells. Cancer Cell.

[23] Yusa K, Tsuruo T (1989). Reversal mechanism of multidrug resistance by verapamil: direct binding of verapamil to P-glycoprotein on specific sites and transport of verapamil outward across the plasma membrane of K562/ADM Cells. Cancer Res.

[24] Hahn HP, Pang M, He J, Hernandez JD, Yang RY, Li LY, Wang X, Liu FT, Baum LG (2004). Galectin-1 induces nuclear translocation of endonuclease G in caspase- and cytochrome c-independent T cell death. Cell Death Diff.

[25] Candé C, Cecconi F, Dessen P, Kroemer G (2002). Apoptosis-inducing factor (AIF): key to the conserved caspase-independent pathways of cell death?. J Cell Sci.

[26] Li LY, Luo X, Wang X (2001). Endonuclease G is an apoptotic DNase when released from mitochondria. Nature.

[27] Won SJ, Wu HC, Lin KT, Yu CH, Chen YT, Wu CS, Huang CY, Liu HS, Lin CN, Su CL (2015). Discovery of molecular mechanisms of lignan justicidin A using L1000 gene expression profiles and the library of integrated network-based cellular signatures database. J Funct Foods.

[28] Asano J, Chiba K, Tada M, Yoshii T (1996). Anti-viral activity of lignans and their glycosides from Justicia procumbens. Phytochemistry.

[29] Gertsch J, Tobler RT, Brun R, Sticher O, Heilmann J (2003). Anti-fungal, anti-protozoal, cytotoxic and piscicidal properties of justicidin B and a new arylnaphthalide lignan from Phyllanthus piscatorum. Planta Medica.

[30] Kinghorn AD, Chin YW, Swanson SM (2009). Discovery of natural product anti-cancer agents from biodiverse organisms. Curr Opin Drug Discov Devel.

[31] Sharpe JC, Arnoult D, Youle RJ (2004). Control of mitochondrial permeability by Bcl-2 family members. Biochimica et Biophysica Acta.

[32] Tait SW, Green DR (2010). Mitochondria and cell death: outer membrane permeabilization and beyond. Nat Rev Mol Cell Biol.

[33] Cregan SP, Dawson VL, Slack RS (2004). Role of AIF in caspase-dependent and caspase-independent cell death. Oncogene.

[34] Prieto JM, Recio MC, Giner RM, Manez S, Massmanian A, Waterman PG, Rios JL (1996). Topical anti-inflammatory lignans from Haplophyllum hispanicum. Z Naturforsch C.

[35] Vanicha V, Kanyawim K (2006). Sulforhodamine B colorimetric assay for cytotoxicity screening. Nat Protoc.

[36] Kajstura M, Halicka HD, Pryjma J, Darzynkiewicz Z (2007). Discontinuous fragmentation of nuclear DNA during apoptosis revealed by discrete “sub-G1” peaks on DNA content histograms. Cytometry Part A.

[37] Moongkarndi P, Kosem N, Kaslungka S, Luanratana O, Pongpan N, Neungton N (2004). Antiproliferation, antioxidation and induction of apoptosis by Garcinia mangostana (mangosteen) on SKBR3 human breast cancer cell line. J Ethnopharmacol.

