# Supplementary material for: Differential modulation of Bax/Bcl-2 ratio and onset of caspase-3/7 activation induced by derivatives of Justicidin B in human melanoma cells A375
Source: Oncotarget. 2017 Oct 6;8(56):95999–6012. doi: 10.18632/oncotarget.21625 (PMC5707076; doi:10.18632/oncotarget.21625)
Supplement: Supplementary file 1 [file oncotarget-08-95999-s001.pdf]

Differential modulation of Bax/Bcl-2 ratio and onset of caspase-3/7 activation induced by derivatives of Justicidin B in human melanoma cells A375

SUPPLEMENTARY MATERIALS

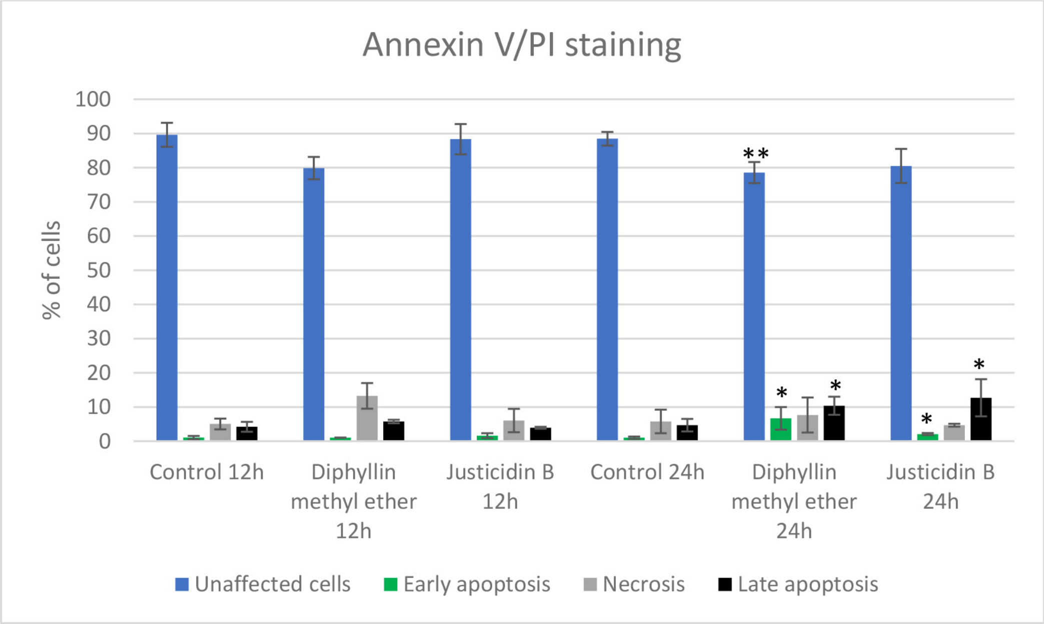

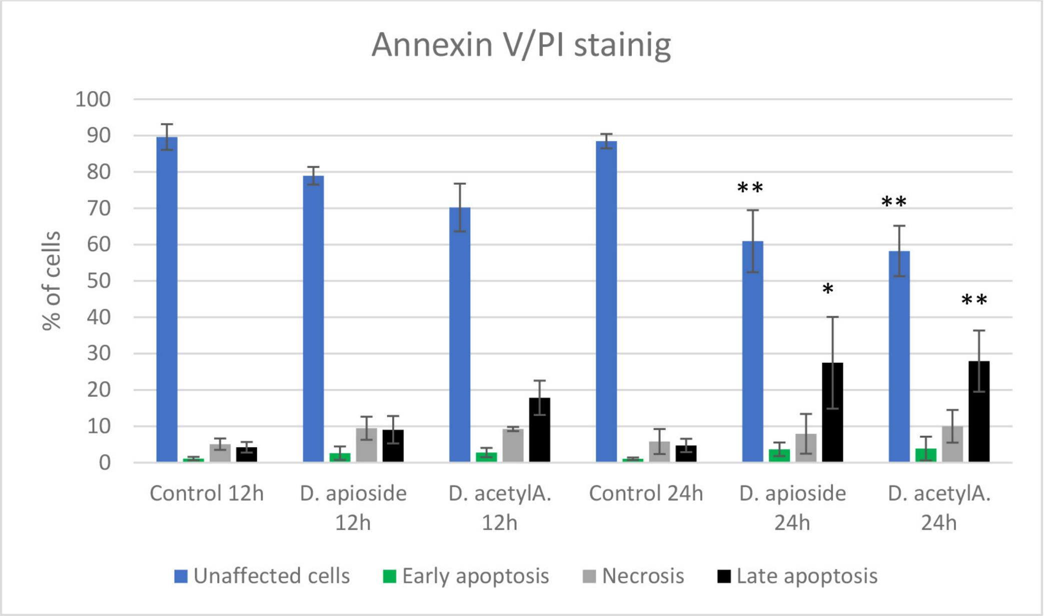

Supplementary Figure 1: Annexin/PI staining results after incubation for 12h vs. 24h. No significant effects were detected at 12h. D. apioside= Diphyllin apioside, D. acetylA= Diphyllin acetylapioside.

Supplementary Table 1: Tabulated numerical results of GI50 values shown in Figure 2

GI_{50} of the compounds (µM)	Paclitaxel	Justicidin B	Diphyllin methyl ether	Diphyllin apioside	Diphyllin acetylapioside
24h	1.32±0.22	>10	>10	>10	>10
48h	0.04±0.03	1.70±0.20	3.66±0.21	0.84±0.06	0.39±0.08
72h	0.0006±0.0009	1.53±1.04	2.11±1.54	0.32±0.30	0.14±0.1

Supplementary Table 2: The flow cytometry cell cycle data values as shown in Figure 3

	Sub-G1	G1	S	G2/M
Control	2.36±1.13	80.19±7.50	15.75±2.76	4.60±2.40
Pac	5.23±0.80	29.90±7.36	39.98±4.77	25.10±3.29
Diphyllin methyl ether	9.67±3.63	60±6.45	26.23±5.05	4.00±2.36
Justicidin B	11.04±2.17	56.32±1.72	26.85±1.48	5.93±1.91
Diphyllin apioside	9.64±3.67	57.41±1.42	26.67±3.30	5.48±3.06
Diphyllin acetylapioside	13.49±5.20	48.23±13.40	28.55±1.40	5.45±3.5

Pac=paclitaxel.

Supplementary Table 3: Annexin/PI staining. Numerical results shown in Figure 4 including those of the positive control Campthotecin

	Control	CPT	Diphyllin methyl ether	Justicidin B	Diphyllin apioside	Diphyllin acetylapioside
Unaffected cells	88.47±1.97	37.61±3.39	78.56±3.10	80.49±4.99	60.93±8.53	58.22±6.94
Early apoptosis	1.03±0.35	7.94±1.80	6.71±3.31	2.10±0.31	3.64±1.87	3.86±3.26
Necrosis	5.79±3.43	10.86±1.89	7.67±5.11	4.69±0.42	7.92±5.45	9.99±4.50
Late apoptosis	4.70±1.82	43.58±4.90	10.38±2.63	12.71±5.45	27.46±12.63	27.92±8.40

CPT=camptothecin.

Supplementary Table 4: Caspase-3/7 activity. Numerical results shown in Figure 6 including those of the positive control Paclitaxel

	Control	Pac	Diphyllin methyl ether	Justicidin B	Diphyllin apioside	Diphyllin acetylapioside
24h	6224±2567	9550±422	15783±4674	4539±4303	6997±3374	7190±4607
48h	21580±7022	157556±48740	61735±21578	10245±9400	94191±43929	63330±24799
72h	98534±582	239282±46144	158530±18343	170327±40249	177859±39789	246298±79122

Pac=paclitaxel.

Supplementary Table 5: Expression of Bax/Bcl-2 proteins. Numerical results for western blot (Bax/Bcl-2 ratio) shown in Figure 6 including those of the positive control Camptothecin

	Bax/Bcl-2
Control	0.03±0.01
CPT	1.11±0.26
Diphyllin methyl ether	1.30±0.58
Justicidin B	0.14±0.01
Diphyllin apioside	0.29±0.04
Diphyllin acetylapioside	0.37±0.09

CPT=camptothecin.
